# A Study of Nutritional and Sensory Qualities of Pea Protein Isolate Beverages with a View to Their Potential Use in Patients with Psychiatric Disorders

**DOI:** 10.3390/foods14172991

**Published:** 2025-08-27

**Authors:** Lasma Plocina, Ilze Beitane

**Affiliations:** Food Institute, Latvia University of Life Sciences and Technologies, LV-3001 Jelgava, Latvia; lasmina-n@inbox.lv

**Keywords:** pea protein isolate, psychiatric disorders, sensory acceptability, nutritional composition, hedonic scale

## Abstract

Patients with mental health disorders often have inadequate intakes of essential amino acids, vitamins, minerals, and fatty acids, which can negatively affect neurotransmitter synthesis, mood, cognitive function, and sensory perception. This study evaluated the nutritional value and sensory acceptability of different flavoured pea protein isolate beverages in 78 patients with schizophrenia spectrum disorders, mood disorders, eating disorders, and depression. The results of the sensory evaluation showed that the sweeter-profile beverages were the best-rated. Nutrient analysis confirmed that the beverages contained important vitamins and minerals, including B_12_, vitamin C, zinc, and magnesium, as well as tryptophan and alpha-linolenic acid, while being low in saturated fat. The results suggest that pea protein isolate beverages are nutrient-rich, well-tolerated, and sensory-acceptable products with high potential as a complementary nutritional solution in mental healthcare.

## 1. Introduction

Psychiatric disorders affect a significant proportion of the world’s population. The World Health Organization estimates that more than 970 million people worldwide suffer from some form of psychiatric disorder, including depression, schizophrenia spectrum disorders, and bipolar disorder [1]. The prevalence of psychiatric disorders has increased significantly in recent decades, affecting quality of life, functioning, and physical health [2]. This group of patients is often diagnosed with comorbidities, such as cardiovascular diseases, metabolic and endocrine disorders, gastrointestinal diseases, as well as functional limitations that make it difficult for patients to absorb adequate amounts of nutrients [3,4,5]. Patients with psychiatric disorders often have poor nutritional intake, which is facilitated by a reduced appetite, increased sensitivity to food texture, and altered taste and smell perception [6]. In addition, many antipsychotic medications, which are integral to therapy, can affect appetite, intestinal motility, and digestive function, making it difficult to consume a complete diet [7]. Consequently, there is a significant risk of developing undernutrition or even malnutrition, especially in patients with depression, schizophrenia, and eating disorders [8,9]. These factors can lead to inadequate absorption of micronutrients, macronutrients, amino acids, vitamins, and essential fatty acids. Studies showed that patients with depression, schizophrenia, and other mood disorders often have reduced levels of tryptophan, tyrosine, serine, glycine, and glutamic acid, which can affect the metabolism of serotonin, dopamine, and gamma-aminobutyric acid (GABA) [10]. Chronic stress and inflammation further impair amino acid bioavailability, increasing the severity of psychiatric symptoms [11].

The B-group vitamins, including B_1_, B_6_, B_12_, and B_9_, are essential coenzymes in the synthesis of neurotransmitters and the methylation processes. Deficiencies in these vitamins are frequently observed in patients with psychiatric disorders, which are associated with impaired homocysteine metabolism, neurodegeneration, and depressive symptoms. Low folate and B_12_ levels are associated with poorer treatment outcomes in depression and schizophrenia [12,13]. Omega-3 fatty acids, especially eicosapentaenoic acid (EPA) and docosahexaenoic acid (DHA), are essential components of brain structure and regulators of inflammation. Their deficiency is associated with a higher risk of depression, bipolar disorder, and even schizophrenia [14]. Omega-3 fatty acid deficiency can increase neuroinflammation by reducing neuronal membrane fluidity. In turn, an adequate omega-3 fatty acid intake can improve brain membrane fluidity, reduce neuroinflammation, and reduce inflammation in the central nervous system [15,16,17,18]. Patients with psychiatric disorders often have deficiencies in minerals such as iron, zinc, magnesium, selenium, and potassium, which impact brain function, oxidative stress, and immune responses [13]. Low vitamin D levels have also been observed, which may be linked to serotonin imbalance [19]. Thus, patients with psychiatric disorders are at a high risk of deficiency of essential vitamins and minerals, which can significantly affect the course of the disease and the results of therapy.

Pea protein isolate is a promising plant-based nutritional component that provides a good profile of essential amino acids, including lysine, leucine, and arginine [20,21]. Unlike many other plant proteins, pea protein isolate also contains a high content of branched-chain amino acids (BCAAs), which may be important in maintaining muscle mass, especially in patients at risk of malnutrition and sarcopenia [20]. Pea protein is also well tolerated, does not contain lactose or gluten, and is therefore suitable for a wide range of patients, including those with dietary restrictions or gastrointestinal sensitivities [20,21].

Despite the growing interest in the use of plant-based protein beverages for nutritional support with various health conditions, there is limited evidence-based data on their efficacy and sensory acceptability in patients with schizophrenia spectrum disorders and mood disorders in the field of mental health [4,22]. Studies show that the correct distribution of nutrients, including a complete supply of amino acids, can have a positive effect on the functioning of neurotransmitters, especially the synthesis of serotonin and dopamine, which in turn can improve mood and behaviour [22,23,24]. The availability of tryptophan, methionine, and histidine, which are often insufficient in the standard diet for this patient group, is essential for the metabolism of B-group vitamins [4,25]. Furthermore, it has been proven that the quality of proteins, not just the quantity, determines their bioavailability and effect on the central nervous system [26].

Although the literature widely describes the problems of nutritional deficiencies in psychiatric patient populations, as well as individual studies having been devoted to the sensory evaluation of functional food products in the general population, there is very limited evidence that simultaneously combines the assessment of both aspects in psychiatric patients. Psychiatric patients often have impaired taste and smell perception, increased sensitivity to texture, and other sensory processing characteristics, which can reduce the acceptance of, and adherence to, new products [27,28]. These disorders can be exacerbated by the deficiencies of certain minerals (e.g., zinc, magnesium, and iron) and vitamins (e.g., B12 and vitamin D), which affect the taste bud function, olfactory receptor activity, and neurotransmitter synthesis [29]. Given the high content of essential amino acids, minerals, and other bioactive substances in pea protein isolate, incorporating it into the diet could optimise nutritional value and sensory acceptability, particularly by developing products with tailored taste and texture properties for psychiatric patients. This combined approach, analysing both nutritional value and sensory acceptability, enables a more accurate evaluation of the product’s effectiveness as a dietary supplement for this patient group.

Taking these aspects into account, the study aimed to evaluate the nutritional value and sensory properties of pea protein isolate beverages in different flavours, considering their potential as a nutritional supplement for patients with psychiatric disorders.

## 2. Materials and Methods

### 2.1. Thermal and Microbiological Safety of Beverage Processing

Pea protein isolate beverages were developed in the laboratory of the Latvia University of Life Sciences and Technologies. The thermal treatment of the beverages was carried out in an autoclave (CFS -50H, TERPA Food-tech, Terrassa, Barcelona, Spain) at 115 °C for 5 min. The specific temperature and time were selected based on the evaluation of the temperature and time inventory performed during the research process, after the assessment of microbiological safety aspects, as well as theoretical and practical aspects of other studies [30]. Compliance with microbiology tolerances was determined by Cabinet of Ministers of the Republic of Latvia Regulation No 461 and European Commission Regulation (EC) No 2073/2005 on microbiological criteria for food [31,32]. After autoclaving, the samples were stored in a refrigerator at +3 °C (±1) for up to one week. The samples were transported at high altitudes without causing large temperature fluctuations. Before serving to patients, the beverages were kept at a room temperature (21 °C ± 1) for up to 20 min.

### 2.2. Summary of the Pea Protein Isolate Beverages Used in This Study

Five different pea protein isolate beverages were developed—lemon (V1), pomegranate–cranberry (V2), blueberry–vanilla (V3), blueberry–lemon (V4), and blackcurrant–apple (V5). Each recipe indicates the amount of ingredients in grams per 200 g of prepared product (Table 1). The ingredients were weighed with a laboratory balance (PS 1000, R2) with an accuracy of ±0.01 g. The recipes are based on four main ingredients:

(1) Organic pea protein isolate from the Netherlands was selected. It is obtained by extracting proteins from yellow peas, and the peas from which it is produced are not genetically modified. To select the most suitable pea protein isolate, the amino acid profiles of organic and commercial pea protein isolates were compared using high-performance liquid chromatography–mass spectrometry (HPLC-MS) [33,34]. The organic pea protein isolate contained a higher essential fatty acid composition. Therefore, it was used as the main protein component in the subsequent beverage production. The juices combined with the pea protein isolate were selected based on data from a study investigating taste acceptability in patients with psychiatric disorders.

(2) Water (solvent);

(3) Nut and hemp powders (cedar—Latvia, walnut—Bulgaria, and hemp—Latvia) were added to supplement the drinks with fatty acids;

(4) Vitamin and mineral powder (United Kingdom) contained B-group vitamins, vitamin C, and D as well as minerals (Zn, Fe, Ca, Mg) provided the beverage with additional nutrients. The flavour profile was achieved by using the appropriate natural, sugar-free fruit, berry juices, and fruit and berry powders.

### 2.3. Laboratory Methods Used in Nutrient Analysis

Nutritional values of all beverage samples were determined at the J.S. Hamilton Baltic laboratory. Validated international methods were used (Table 2).

### 2.4. The Sensory Evaluation of the Beverages

The research design was qualitative, conducted from 16 May 2025 to 22 May 2025 at the NPVC (National Centre of Mental Health, State Ltd.), Riga, Latvia.

The inclusion criteria for patients were as follows: (1) Patients with schizophrenia spectrum disorders, mood disorders, eating disorders, or depression were hospitalised at the NPVC, taking antipsychotic medications or antidepressants for at least one year. (2) Patients were older than 18 years and legally competent. (3) Patients were able to eat independently without assistance from others. (4) Patients did not have a nasogastric tube or PEG (percutaneous endoscopic gastrostomy). (5) Patients had not smoked in the last hour. (6) To ensure the reliability of the sensory evaluation, only participants with preserved cognitive capacity, the ability to understand instructions, and those able to provide reliable responses were included. All potential participants were screened and approved by their treating psychiatrist before being included in this study.

Patient exclusion criteria were as follows: (1) All possible diseases that may additionally affect taste changes are excluded—type 1 and 2 diabetes, oncological diseases, COVID-19 or other viral diseases, chronic kidney disease, cardiovascular disease, HIV/AIDS, and vitamin B_12_ and zinc deficiency, if determined. (2) All possible medications that may affect taste changes are excluded, except for antipsychotic medications and antidepressants, angiotensin-converting enzyme inhibitors, metformin, antibiotic use, antifungal medications, antihistamines, diuretics, and anticonvulsant medications. (3) Allergy to legumes (beans, peas), nut powders (walnuts), grapefruit, lemon, blackcurrants, blueberries, apples, beets, cranberries, ascorbic acid, currants, pomegranates, vanilla extract, or inulin (plant reserve polysaccharide).

This study included participants (*n* = 78) with diagnoses that included schizophrenia spectrum disorders, mood disorders, eating disorders, and depression. Potential study participants were invited to participate in this study via an information letter. Patient participation was previously coordinated with the heads of the NPVC inpatient departments and treating physicians, taking into account the above-mentioned inclusion and exclusion criteria. Study participants were introduced to the composition of the pea protein isolate beverages. Each study participant was given a 5-point hedonic scale (5—like very much, 4—like, 3—neither like nor dislike, 2—dislike, 1—dislike very much), ISO 4121; ISO 6658 [57,58], as well as instructions for its completion. Each study participant was given five different pea protein isolate beverage samples (each sample, 25 mL, in a clean, disposable cup). Each beverage sample was assigned a unique code. The planned study time was up to 25 min per patient. After each sample, it was recommended to neutralise the taste, for which water was used. All samples were evaluated during one session. However, to ensure the desired number of patients, several sessions were held. Session times were previously coordinated with the heads of the NPVC inpatient departments to minimise the impact on the patients’ daily routine.

### 2.5. Statistical Analysis and Evaluation of Sensory Data

Statistical processing was performed using RStudio (version 4.3.1). For each of the five beverages (V1–V5), nutrient values (amino acids, fatty acids, vitamins, and minerals) were calculated per 100 g and further normalised to 200 g for comparison with the recommended daily allowances (RDA) as defined in the WHO (World Health Organization), EFSA (European Food Safety Authority), and USDA (United States Department of Agriculture) guidelines for adults. To visualise the essential amino acid content, a heatmap was created showing the amino acid content (% of WHO recommendations per 100 g of product) separately for each beverage sample. Eight essential amino acids were included in the analysis: valine, tryptophan, threonine, phenylalanine, methionine, lysine, leucine, and isoleucine. For multivariate assessment, principal component analysis (PCA) was conducted to identify the dominant variables influencing sample separation. The results are presented as biplots, showing the beverage samples (V1–V5) and the dominant nutrient vectors projected along the principal components (PC1 and PC2). All data are presented as means ± standard deviations (SD), calculated from *n* = 5 independent repetitions per sample. The following R packages were used for data analysis and visualisation: ggplot2, gplots, reshape2, factoextra, and PMCMRplus.

### 2.6. Ethical Considerations

This study was conducted by the Declaration of Helsinki, and permission was obtained from Riga Stradiņš University on 22 April 2025, 2-PĕK-4/680/2025, and from the National Centre of Mental Health (NPVC), State Ltd. (Riga, Latvia), on 16 May 2025, No. NPVC/14-02/25/4789. Informed consent was obtained from all participants involved in this study.

## 3. Results

### 3.1. Nutritional Value Analysis of Pea Protein Isolate Beverages 

Nutrient distribution among five pea protein isolate beverages and a comparison of their results with the RDI (recommended daily intake) for an adult, as per the Ministry of Health of the Republic of Latvia, per 200 g of product, is shown in Table 3.

The lemon flavoured beverage (V1) provides 7.0% of the daily energy requirement, with low levels of sugar (12.8%) and carbohydrates (2.0%). Its fibre content (4.8%) is moderately good, while the protein content reaches 18.6 g or 37.2% of the RDI, indicating a high protein concentration. The pomegranate–cranberry flavoured beverage (V2) provides 8.1% of the energy, with a high sugar content of 28.8% of the RDI. The fibre content (8.8%) and protein content (18.8 g, 37.6%) make it a potentially good addition to a diet where a high protein intake is required, but the sugar load should be taken into consideration. The blueberry–vanilla flavoured beverage (V3) has the highest proportion of sugar, at 53.6% of the RDI per 200 g serving. The protein content is 19.8 g (39.6% of the RDI), but the fibre content is relatively low (4.0%). The use of this beverage should be limited due to its high sugar content. However, it provides a significant amount of protein. The blueberry–lemon flavoured beverage (V4) provides 8.9% of the daily energy requirement and 9.2% of carbohydrates. The sugar content is 28.8%, fibre is 5.6%, and protein is 18.0 g, or 36.0% of the RDI. Overall, there is a balanced profile, except for sugar concentration. The blackcurrant–apple flavoured beverage (V5) stands out with the highest fibre content—11.2% of the RDI, and the highest protein content—20.8 g or 41.6% of the RDI. The energy value is 9.7% of the daily energy requirement, and the sugar content is 41.6% of the RDI. The product is nutritionally valuable, but it is not suitable for regular use without regular sugar monitoring. The highest sugar content observed among the beverages was 6.7 g per 100 g, confirming that the developed products can be regarded as containing a relatively low amount of sugar.

### 3.2. The Amino Acid Composition of Pea Protein Isolate Beverages

Pea protein isolate is characterised by a relatively balanced amino acid composition, including both essential and non-essential amino acids, which makes it a promising plant-based protein source for the development of specialised nutritional and therapeutic food products. The amino acid profile of five different pea protein isolate beverages, expressed per 100 g of dry matter, is summarised in Table 4.

The dominant amino acids in all beverages are glutamic acid up to 1.81 g 100 g^−1^, aspartic acid 1.20–1.14 g 100 g^−1^, and arginine 0.733–1.01 g 100 g^−1^. The blackcurrant–apple flavoured beverage (V5) showed the highest total amino acid content, especially standing out with the highest concentrations of lysine 0.729 g 100 g^−1^, leucine 0.861 g 100 g^−1^, phenylalanine 0.551 g 100 g^−1^, and tryptophan 0.105 g 100 g^−1^. The tryptophan content, which is essential for serotonin synthesis, reaches the highest value in the blackcurrant–apple flavoured beverage (0.105 g), but the lowest in the pomegranate–cranberry flavoured beverage (0.085 g). Histidine, methionine, cysteine, and tryptophan, as essential amino acids, are found in all beverages in relatively uniform concentrations. Taking this profile into account, it can be concluded that pea protein isolate beverages have the potential to provide a valuable amino acid composition, which could be particularly important for patients with mental health disorders, as the balance of proteins and amino acids can affect neurotransmitter synthesis and psychoneurological function [29,61].

**Figure 1:**
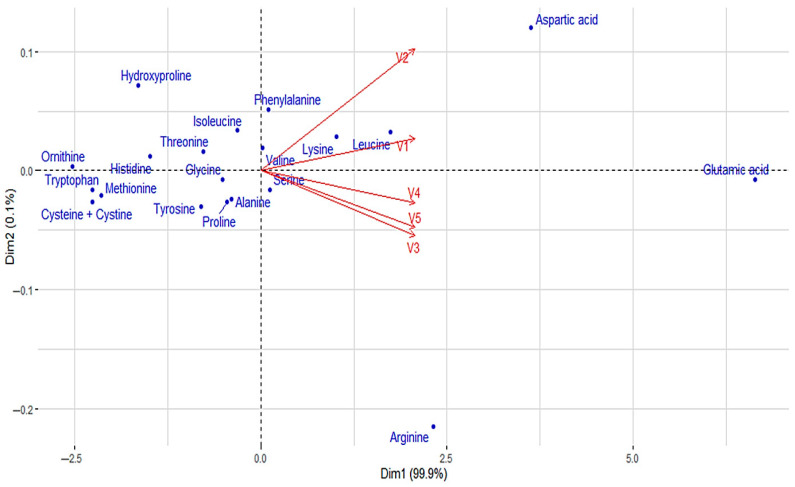
The amino acid profile visualisation of pea protein isolate beverages with a biplot.

Principal component analysis (PCA) was used to visually represent and compare the amino acid profiles of five different pea protein beverages (V1–V5). The graph in Figure 1 plots both beverages (vectors) and amino acids (dots) based on their relative contributions to the first two principal components (Dim1 and Dim2), which together explain 99.9% and 10.1% of the variance in the data.

The results showed marked differences between the beverages, where the blackcurrant–apple flavoured beverage (V5) is particularly rich in glutamic acid, aspartic acid, and arginine, which dominate the PCA plot on the right side, moving away from the other points. The blueberry–vanilla flavoured beverage (V3), blueberry–lemon flavoured beverage (V4), and blackcurrant–apple flavoured beverage (V5) cluster closer together, reflecting similar amino acid contents (serine, lysine, leucine, and valine), but differ slightly in intensity. The lemon flavoured beverage (V1) and the pomegranate–cranberry flavoured beverage (V2) stand out with a relatively lower amount of dominant amino acids, positioning themselves closer to the PCA centre point. Amino acids such as hydroxyproline, tryptophan, methionine, and histidine are located closer to the intersection, indicating their equal occurrence in all beverages. The analysis helps identify which beverages are particularly suitable for the intake of specific essential amino acids, as well as indicating the possible need for supplementation in other cases.

**Figure 2:**
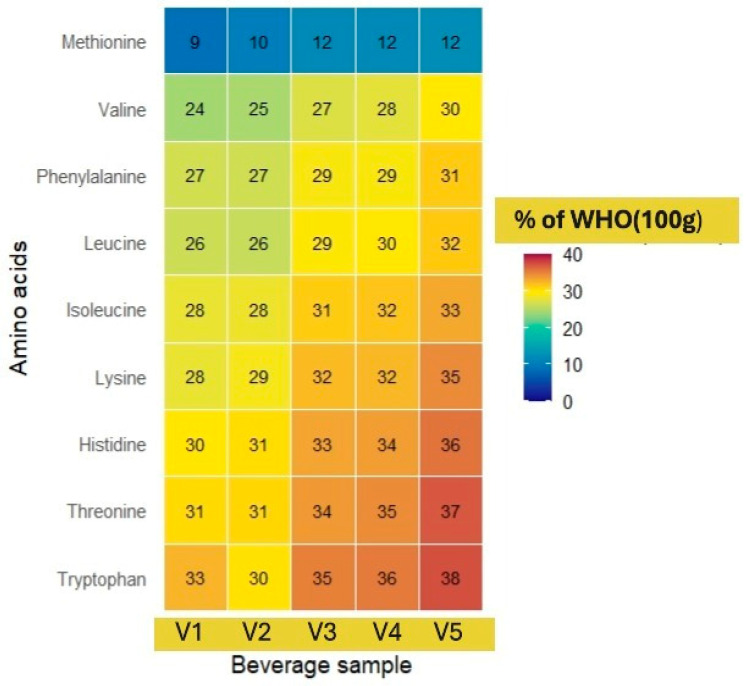
The composition of essential amino acids of pea protein isolate beverages compared to the WHO recommendations per 100 g of product.

The heat map (Figure 2) depicts the content of eight essential amino acids (valine, tryptophan, threonine, phenylalanine, methionine, lysine, leucine, and isoleucine) in five pea protein beverage samples compared to the WHO recommendations for adults (per 100 g of product). These recommendations are based on standards defined for an adult with a body weight of 70 kg [62].

The heat map visually highlights the differences in amino acid concentrations in the five developed beverages (V1–V5). In all beverage samples, the highest relative values were observed in the contents of histidine, tryptophan, and threonine, reaching as much as 37% of the WHO’s recommended daily intake per 100 g of product. Lysine, isoleucine, and leucine also showed high contents in all beverages, remaining in the range of 28–35%. In turn, the content of methionine in all five beverages is significantly lower than that of the other amino acids, with relative values of only 9–12% of the RDI, confirming its status as the limiting amino acid in pea protein isolate. Among the beverages, relatively higher values are observed in the blackcurrant–apple flavoured beverage (V5), especially in the contents of tryptophan (38%) and histidine (36%), which can potentially affect mood regulation and neurotransmitter synthesis [62]. Overall, the heat map reveals similar essential amino acid profiles among all beverages, with small but clinically significant differences in specific components.

### 3.3. The Fatty Acid Profile of Pea Protein Isolate Beverages

The analysis revealed that 200 g of the product yields significantly different amounts of fatty acids, depending on the flavour of the beverage (Table 5). The highest proportion of the recommended daily allowance (RDA) was observed in the polyunsaturated fatty acid (PUFA) group—up to 16% (sample V1), which is positive, considering the importance of PUFA in the functioning of the nervous system. Similarly, α-linolenic acid (ALA), a plant-based source of omega-3 fatty acids, which is considered an essential supplement for patients with an increased need for omega-3 fatty acids, reached up to 30.8% of the RDA (samples V1 and V2). In turn, SFA and trans fatty acids showed very low proportions (around 1.8–2.7% and less than 1%, respectively), which is desirable for nutritional prevention, particularly in patients with depression or other inflammation-related psychiatric disorders. Although the content of MUFA and omega-6/omega-9 fatty acids is modest, it could still provide some biological functionality. The content of trans fatty acids was consistently low (≤0.2 g/100 g), not exceeding the WHO-recommended level (<0.5% E%). It is important to emphasise that α-linolenic acid (ALA, C18:3n-3) was also identified in all drinks. This essential omega-3 fatty acid serves as a precursor for the synthesis of EPA and DHA in the human body.

The ALA content in the samples was 0.1–0.20 g 100 g^−1^ DW, which, considering the recommended daily intake by EFSA of approximately 2 g/day, constitutes approximately 5% of the required amount in one serving of 200 g of beverage. Overall, beverages can be considered a safe source of fatty acids that could be relevant to brain function from a nutritional perspective.

### 3.4. Mineral and Vitamin Composition of Pea Protein Isolate Beverages

The pea protein isolate beverages provided high amounts of micronutrients, especially vitamins C, B_12_, B_7_ (biotin), and B_9_ (folate) (Table 6). The amount of biotin (B7) in all beverages is between 105.7% (sample V1) and 143.0% (sample V5). Folic acid (B_9_) is also provided in more than 120% of the RDA in several variants. Vitamin B_12_ in all beverages exceeds 700%, with a maximum value of 816.7% in the blackcurrant–apple beverage (V5). These indicators are significant for psychiatric patients, where deficiencies of these vitamins are often observed. Zinc (Zn) content ranges from 150.7% (sample V3) to 188.5% (sample V5). Magnesium (Mg) ranges from 94.0% (sample V2) to 106.7% (sample V1). Selenium (Se) is provided in the range from 58.2% to 65.5%, and iron (Fe) between 67.0% and 75.2%. The amount of calcium (Ca) is lower, ranging from 40.0% to 45.0%. Sodium (Na) in all beverages remains below 8.3% of the daily dose, which may be valuable for patients with hypertension.

In general, these beverages are characterised by their exceptionally high vitamin content, particularly B-group vitamins and vitamin C. In many cases, this exceeds the RDI by 500–800%. They have the potential to serve as dietary supplements for psychiatric patients at high risk of micronutrient deficiency.

### 3.5. The Sensory Evaluation of Pea Protein Isolate Beverages

**Figure 3:**
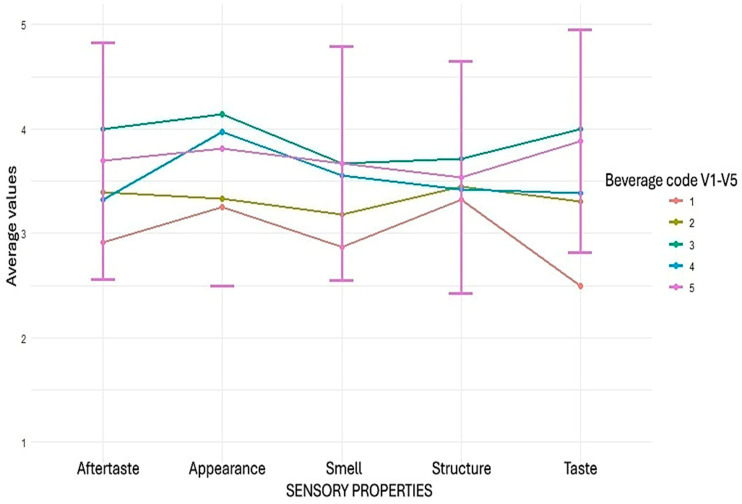
Sensory evaluation of pea protein isolate beverages with different flavours among 78 patients.

The sensory evaluation results revealed significant differences between the five developed pea protein beverages (V1–V5), which were evaluated in five attributes using a 5-point hedonic scale (1 = “very dislike”, 5 = “very like”). Overall, the highest ratings were obtained by the blueberry–vanilla flavoured beverage (V3) and the blackcurrant–apple flavoured beverage (V5), which were consistently rated higher than the other samples, especially in terms of taste and visual appearance (Figure 3).

In terms of taste profile, the blueberry–vanilla flavoured beverage (V3) was rated highest (4.00 ± 0.98), followed by the blackcurrant–apple flavoured beverage (V5) (3.88 ± 1.09). In contrast, the lemon flavoured beverage (V1) received a significantly lower rating (2.50 ± 1.30), which coincides with the pronounced decline depicted in the line graph. Aftertaste ratings were dominated by the blueberry–vanilla flavoured beverage (4.00 ± 1.07) and the blackcurrant–apple flavoured beverage (3.69 ± 1.13), while the lemon flavoured beverage (2.91 ± 1.31) had the lowest rating and the most significant standard deviation among the beverages. In the appearance assessment, the blueberry–vanilla flavoured beverage stood out with the highest average (4.14 ± 0.94), leaving a significant distance to the lemon flavoured beverage (3.26 ± 0.95) and the pomegranate–cranberry flavoured beverage (3.33 ± 1.05). Still, the texture ratings were relatively even among all beverages (3.32–3.72), with a slight advantage for the blueberry–vanilla flavoured beverage (3.72 ± 1.06). In terms of aroma, the blueberry–vanilla flavoured beverage and the blackcurrant–apple flavoured beverage (both 3.67) were rated higher than the other beverages, while the lemon flavoured beverage again received the lowest rating (2.87 ± 1.04), confirming the overall lower acceptability of this sample. The sensory evaluation data were analysed using the Kruskal–Wallis test to assess differences among the four beverage samples across five sensory attributes. The analysis revealed statistically significant differences in appearance (H = 50.02; *p* < 0.00000000036), aroma (H = 32.88; *p* < 0.00000126), taste (H = 67.57; *p* < 0.00000000000074), and aftertaste (H = 35.40; *p* < 0.0000003849) scores. In contrast, there were no significant differences in texture ratings between samples (H = 6.03; *p* = 0.197).

These findings suggest that, except for texture, the beverage formulations differed significantly in most sensory attributes. The results provide a solid basis for further optimisation of beverage composition and processing technology, particularly with regard to enhancing taste, visual appearance, aroma, and aftertaste.

## 4. Discussion

The pea protein isolate beverages analysed in the study showed great promise as a functional dietary supplement for patients with psychiatric disorders. However, further research is needed to bring the beverages to market. The average energy value of the beverages was approximately 70–85 kcal per 100 g, which provides 7–10% of the recommended daily energy intake for adults, which is 2000 kcal/day [80]. The use of the beverage may be significant for patients with psychiatric disorders, as these patients often experience reduced appetite, various nutrient deficiencies, and difficulty consuming a varied or complexly prepared diet [7,81,82]. The beverages were low in total fat, relatively high in fibre, and contained low levels of added sugar. Some beverages contained more than 5 g of sugar per 100 g, but most of the sugar comes from natural juices rather than refined added sugar. Given that dietary sweetness can significantly influence the acceptability of a product’s taste, a modest increase in natural sugar content may significantly improve product use in patients with psychiatric disorders. Although the scientific literature has identified potential risks between excessive sugar consumption and worsening symptoms of psychiatric disorders, moderation of natural sugar sources and the balanced nutritional profile of the beverage significantly mitigate this effect [83,84,85,86]. Thus, the composition of the beverages provided a good compromise between nutritional value and sensory acceptability. The protein content of the beverage, 9.0 g 100 g^−^^1^, is high for a plant-based beverage. The EFSA recommends a daily intake of approximately 0.83 g of protein per kg of body weight for a healthy adult, which would be 58 g for a 70 kg body weight [66]. Consuming 200 g of pea protein isolate beverage can provide approximately 31% of the daily protein requirement. Additionally, the quality of the protein in this product was enhanced by a complete amino acid profile. The developed pea protein isolate beverages had higher nutritional values, with protein content ranging from 9.0 to 10.4 g 100 g^−^^1^ and dietary fibre ranging from 0.5 g to 1.4 g 100 g^−^^1^, compared to studies where soy, rice, oat, and almond proteins were analysed, in which protein content ranged from 0.42 to 2.78 g 100 g^−^^1^ and fibre content was lower, from 0.4 to 0.75 g 100 g^−^^1^, and also provided higher bioavailability after heat treatment [87,88,89].

Amino acids are essential for maintaining mental health because they serve as precursors for neurotransmitters (e.g., tryptophan for serotonin and tyrosine for dopamine) and influence brain function, mood swings, stress, and metabolism regulation [90]. Patients with psychiatric disorders, such as depression, schizophrenia, or anxiety, often have amino acid deficiencies or impaired metabolism [61,91]. The analysis of essential amino acids showed that the highest values were observed for glutamic acid 1.81 g 100 g^−^^1^ g DW, which dominated all beverages. Similarly, arginine, which reached up to 1.01 g 100 g^−^^1^ DW, was significantly higher than in other plants as well as in oat or almond beverages—<0.2 g 100 g^−^^1^ [92]. Arginine has neuroprotective and circulatory effects, which may be particularly important in cases of cognitive impairment [84,93]. Arginine is a substrate for nitric oxide synthesis, which improves cerebral blood flow and neuroplasticity. In studies of patients with schizophrenia, an arginine-rich diet (>6 g/d) increased N-acetyl-aspartate levels in the frontal cortex [94]. Thus, pea protein isolate beverages can potentially support neurochemical balance as an adjunct to therapy. Pea protein isolate beverages (V1–V5) provided a significant amount of essential amino acids, which are essential for the functioning of the central nervous system. Overall, the highest total amount of essential amino acids was observed in the blueberry–vanilla flavoured beverage (V3), followed by the pomegranate–cranberry flavoured beverage (V2) and the blackcurrant–apple flavoured beverage (V5). This profile suggests that pea protein beverages could be suitable for patients with psychiatric disorders, who are often found to have an insufficient intake of essential amino acids, protein metabolism disorders, as well as imbalances in neurotransmitter synthesis associated with the flow of essential amino acids [95,96]. Leucine activates cell-signalling pathways and promotes the expression of brain-derived neurotrophic factors, thereby supporting synaptic plasticity and mood regulation. There is also evidence that leucine may stimulate muscle protein synthesis and improve muscle growth in healthy individuals and those with specific diseases, such as cancer or age-related muscle loss [97]. Leucine is found in both animal and plant foods, and its content can be negatively affected by preservation; therefore, it is essential to select an appropriate processing method [98]. The leucine content reached 0.861 g/100 mL (87.9% of the recommended daily intake, RDI, per 70 kg body weight), while in other samples, it ranged from 0.712 g (72.7%, sample V2) to 0.814 g (83.1%, sample V4). Lysine, which reduces anxiety by acting as a serotonin 5-HT receptor antagonist [89], was most abundant in the blackcurrant–apple flavoured beverage (0.729 g/100 mL, 86.8% of the RDI) compared to lower levels in V1–V4 (70.5–81.2%) [99]. The isoleucine content in the blackcurrant–apple flavoured beverage (0.465 g/100 mL, 66.4%) also surpassed that in other beverages (ranging from 56.4% in sample V1 to 63.6% in sample V4). This branched-chain amino acid plays a role in glucose uptake, energy production, and neural function [100]. Regarding phenylalanine, the blackcurrant–apple flavoured beverage (V5) again led with 0.551 g/100 mL (56.2%), followed by the blueberry–vanilla flavoured beverage (V3) and the blueberry–lemon flavoured beverage (V4). Given that phenylalanine is a precursor for dopamine and norepinephrine, its presence supports attention, motivation, and affective stability [101]. Threonine, essential for myelin sheath formation and neural transmission, was present in the blackcurrant–apple flavoured beverage (V5) at 0.390 g/100 mL (79.6%), slightly exceeding values in other samples (65.9–74.1%) [102]. Methionine, a methyl donor involved in SAMe synthesis and epigenetic regulation, was again most concentrated in the blackcurrant–apple flavoured beverage (0.541 g/100 mL, 77.3%), with other beverages providing concentrations ranging from 63.4% to 72.4% [103]. Tryptophan is an essential amino acid that is essential for the synthesis of serotonin and melatonin. Its deficiency is associated with depression, anxiety, and sleep disorders [104]. Tryptophan, essential for serotonin and melatonin synthesis, was found in the blackcurrant–apple flavoured beverage (V5) at 0.105 g/100 mL, covering 37.5% of the WHO/FAO (Food and Agriculture Organization) RDI for a 70 kg adult. Other samples were provided at between 30.4% and 35.6%. Importantly, many plant-based beverages (such as almond, rice, and oat beverages) lack sufficient tryptophan, making pea protein isolate a particularly relevant dietary source [105].

The amino acid profile of the beverages demonstrates that they meet nearly all the amino acid requirements, as per the WHO/FAO [106]). The amino acids in these beverages must come from a plant origin, specifically pea protein isolate. Plant proteins tend to have a lower biological value than animal proteins; however, appropriate combinations and processing methods, such as heat treatment in an autoclave, can improve their digestibility and bioavailability [97]. A pea protein isolate beverage is a good alternative for patients with dietary restrictions, offering a greater preference for plant-based nutrition. It can be concluded that the amino acid profile of pea protein isolate beverages was balanced and had high nutritional value. These beverages may provide valuable nutrients that help maintain brain function and could be used as a complementary dietary option for individuals with mental health conditions. For the efficient metabolism of many essential amino acids, such as tryptophan, phenylalanine, and methionine, enough B-group vitamins are required, which is also proven by the additional enrichment of beverages with B-group vitamins. The conversion of tryptophan to serotonin is dependent on vitamin B_6_, while the conversion of phenylalanine to dopamine requires B_6_, folate (B_9_), and B_12_. Similarly, the methylation pathway of methionine to S-adenosylmethionine (SAMe), an essential neurotransmitter co-regulator, is only possible in the presence of B-group vitamins [107,108]. These associations suggest a complex interaction between amino acid availability and micronutrients; therefore, in addition to an EAA-rich beverage, the inclusion of vitamins B_6_, B_12_, and B_9_ in the diet of patients with psychiatric disorders would also be recommended to ensure the maximum efficiency of neurotransmitter biosynthesis [95].

Pea protein isolate beverages were found to contain significant amounts of vitamins and minerals that may contribute to mental health in patients with schizophrenia spectrum and mood disorders. All pea protein isolate beverages were found to contain high levels of B-group vitamins, which are essential for neurotransmitter synthesis and homocysteine regulation, reducing the risk of depression and cognitive impairment [12]. The concentration of vitamin B_12_ in the beverages provided more than 50% of the recommended daily intake, according to the EFSA recommended intake for adults, of 4 μg (0.004 mg) per day [109]. The WHO/FAO recommends 2.4–2.8 μg per day as the minimum required amount for adults [110]. Neither the EFSA nor the WHO has set an upper tolerable dose, as no toxic effects have been found even at very high doses (0.5–1.0 mg/day) of B_12_ from food or supplements [109,110]. Vitamin B_12_ is essential for a normal nervous system function and psychological function. Deficiency can contribute to depression, cognitive decline, memory impairment, and even psychotic episodes. Studies have shown that an adequate intake of B_12_ can improve mood, reduce anxiety, and stabilise energy levels. B_12_ participates in methylation processes (along with folate and B_6_), which are necessary for the synthesis of neurotransmitters (dopamine, serotonin, norepinephrine) [110]. The pea protein isolate beverages were high in vitamin C (102–1118% RDI), which may reduce oxidative stress. In meta-analyses, vitamin C supplementation (≥500 mg/d) and vitamin D supplementation at 5000 IU/d showed a small but statistically significant reduction in depressive symptoms [111].

Zinc content (6.0–7.5 mg; 150–189% RDI) is essential for the modulation of neurotransmitter receptors; 25 mg Zn/d reduced BDI-II (second part of the Beck Depression Inventory) by five points in a 12-week study [112]. Regarding the zinc content of the developed beverages, the highest concentration measured was 15.08 mg in one of the beverages (V5), which is below the Tolerable Upper Intake Level (UL) of 25 mg/day for adults established by the European Food Safety Authority. The recommended daily intake for adult men in Europe is between 9.4 and 16.3 mg, and for adult women, it is between 7.5 and 12.7 mg. As these beverages are intended to complement the daily diet rather than serve as the sole source of zinc, this concentration does not exceed safe intake thresholds. However, prolonged consumption in combination with a zinc-rich diet could bring the total intake closer to the UL. Exceeding this level may lead to reduced copper absorption, altered immune function, and gastrointestinal disturbances. Therefore, this product should be used under medical supervision, particularly by patients with long-term dietary supplementation needs [113,114].

Iron (5.5–6.2 mg; 68–75% RDI) is a cofactor for dopamine hydroxylase; clinical data suggest that iron supplementation improves attentional control in patients with ADHD (attention deficit hyperactivity disorder) [100].

Magnesium (Mg) levels were 94–106% RDI, and Mg citrate (248 mg/d) for six weeks reduced MDD (major depressive episode) symptoms by 22% [115].

Selenium (58–65% RDI) is associated with a better cognitive function; 2 × 200 µg/d improved MOCA (screening for coccygeal disorders) scores in elderly depressed patients [116].

The calcium content in pea protein isolate beverages was up 40%, which was higher than in soy and oat beverages, and helps reduce the risk of osteopenia caused by antipsychotic drugs [117]. This composition of minerals and vitamins complies with EFSA recommendations and is suitable for patients with an increased neuropsychological risk [80].

A comparison of pea protein isolate beverages (V1–V5) reveals significant differences in their fatty acid composition, which may affect their suitability for patients with psychiatric disorders. Several beverages provide functionally substantial amounts of omega-3 fatty acids, especially α-linolenic acid (ALA), the content of which in the lemon flavoured beverage (V1), the pomegranate–cranberry flavoured beverage (V2), and the blackcurrant–apple flavoured beverage (V5) exceeded 0.2 g/100 g, representing more than 30% of the recommended daily intake per 200 g of product. Similarly, the EPA and DHA content of 0.1 g/100 g is unusually high for 100% plant-based products, which may contribute to supporting brain health even in the absence of animal-derived fatty acids. The pomegranate–cranberry flavoured beverage (V2) is particularly noteworthy, with an optimal omega-6/omega-3 ratio (4:1), low saturated fatty acids (1.8 g of total fat), and a balanced monounsaturated and polyunsaturated fatty acid profile. This is consistent with the recommendations of the EFSA and the WHO, where the recommended daily intake of saturated fatty acids is 10% of E%, omega-6 is 4−6% of E%, and ALA is 1.1−1.6 g/d [63,64]. This composition is particularly suitable for patients taking second-generation antipsychotic medications and who are at an increased risk of metabolic syndrome [118]. From a clinical perspective, the presence of omega-3 (ALA, EPA, DHA) and omega-9 (oleic acid) fatty acids promotes synaptic plasticity, reduces inflammatory markers, and promotes an increase in BDNF (brain-derived neurotrophic factor) levels, which is particularly important in cases of depression, cognitive impairment, and schizophrenia spectrum disorders [119]. Oleic acid additionally stimulates the mTOR pathway, which affects neuronal growth, myelination, and emotional regulation mechanisms [120]. Beverages with a lower ALA content (the blueberry–vanilla flavoured beverage and the blueberry–lemon flavoured beverage) may be suitable for patients who additionally receive omega-3 fatty acid supplements or who require a lower fatty acid load (e.g., in cases of more severe somatic comorbidities). These observations confirm that the fatty acid composition included in pea protein isolate beverages—especially the presence of α-linolenic acid (ALA), EPA/DHA, and oleic acid—may support neuropsychological health by reducing inflammation and promoting synaptic plasticity. The use of beverages with a low saturated fatty acid content and a high polyunsaturated profile is substantial for patients at an increased risk of metabolic syndrome and cognitive impairment. Patients with psychiatric disorders, especially schizophrenia and mood disorders, often experience changes in their sense of taste and smell [121]. These changes may be associated with neurotransmitter imbalances, particularly changes in dopamine and serotonin metabolism, as well as long-term drug therapy that can affect the function of taste receptors [121,122]. Sensory disorders can cause taste distortion (dysgeusia), reduced sensitivity (hypogeusia), or even complete loss of taste (ageusia), which in turn significantly affects food intake and eating habits [121]. In such cases, the choice of flavours in functional food products is crucial—they can both promote and hinder the daily use of the product. The pea protein isolate beverages developed in this study were offered in five different flavours, based on potential patient preferences and previous research, which suggests that citrus and berry flavours may be particularly acceptable to people with altered sensory perception [121].

Sensory evaluation using a five-point hedonic scale showed that beverages with a stronger citrus note (e.g., lemon and blueberry–lemon) were rated less favourably, especially in terms of aftertaste and texture. The lemon flavour beverage received the lowest overall score, especially in terms of aftertaste (average 2.1 points), suggesting a possible overly pronounced or unpleasant perception of acidity by patients. In contrast, beverages with a higher sweetness note (e.g., the blackcurrant–apple and the blueberry–vanilla flavoured beverages) were rated significantly higher, especially in the taste and aftertaste categories. The blueberry–vanilla flavoured beverage received an average score of 4.1 for taste and 4.0 for aftertaste, indicating its sensory acceptability. This finding is consistent with previous literature reports suggesting that patients with psychiatric disorders tend to prefer sweet, less intense, and simpler taste qualities [121,123]. These observations confirm that the sensory properties of beverages are a decisive factor in product acceptance in this target group. Based on the data, it can be concluded that beverages with a sweeter, smoother, and more balanced taste profile are potentially more suitable for nutritional supplementation in patients with psychiatric disorders. However, sour taste nuances may reduce motivation to consume them. The results of this study provided insights into the nutritional composition and sensory acceptability of pea protein isolate beverages (V1–V5), highlighting their potential as a complementary dietary option for patients with psychiatric disorders. A detailed nutritional analysis combined with a sensory acceptability evaluation showed that several beverages (especially V1, V2, and V5) contain significant amounts of essential amino acids, omega-3 fatty acids (ALA, EPA, DHA), trace elements, and B-group vitamins. According to EFSA and WHO recommendations, these nutrients are important for maintaining nutritional adequacy and supporting neuropsychiatric health. In addition, considering the positive sensory evaluation in the target group of patients, beverages with a sweeter and milder taste profile were particularly highly rated, which may promote their regular use, as patients with taste disorders often have reduced motivation and appetite. Thus, the study design simultaneously addresses both nutrient deficiencies and reduced sensory acceptability, which are common problems in mental healthcare.

This study marks a significant step forward in the individualisation of clinical nutrition, offering an innovative plant-based alternative for patients with complex metabolic and neuropsychiatric profiles. Further studies are needed to evaluate the long-term effects of these beverages, as well as their ability to modulate specific biomarkers (e.g., albumin, glucose, cholesterol fractions, B_12_, iron, C-reactive protein, BDNF, or inflammatory cytokines) in patients with depression, ADHD, or schizophrenia spectrum disorders. A broad spectrum of nutrients is what the beverages were designed to supply, but it is important to consider some potential limitations. For example, the methionine content of some formulations did not meet the WHO/FAO recommended daily intake, which could limit the product’s use as a sole protein source in the long term. Similarly, although the nut-derived ingredients improved the fatty acid profile, their potential to cause allergies may limit their use in specific patient groups. Sensory evaluation revealed differences between beverages, but in all cases, a level of acceptability was achieved. These differences suggest that further product development would require the refinement of flavour nuances to meet the preferences of the target group. A limitation of this study that should be mentioned is that all participants were recruited from a single mental health centre, which may limit the generalisability of the findings. However, strict inclusion and exclusion criteria were applied to ensure that only participants with preserved cognitive abilities, the capacity to understand instructions, and the ability to provide reliable responses were included. All potential participants were evaluated and approved for participation by their treating psychiatrist. Clinical trials with objective evaluation criteria would be needed to assess the potential impact of these beverages on patient health and psychological functioning. It is recommended to include the biochemical parameters listed above, as well as appetite assessment, changes in dietary intake, and general functional status, such as body mass index, muscle mass, and fat mass.

## 5. Conclusions

The blackcurrant–apple flavoured beverage (V5) showed the highest concentration of essential amino acids (especially tryptophan and lysine), as well as the best sensory acceptability profile. It may therefore be considered as a complementary dietary option with potential relevance for patients with psychiatric disorders. In addition, the beverages contained a significant amount of polyunsaturated fatty acids, including plant-based omega-3 fatty acids (α-linolenic acid), as well as substantial amounts of B-group vitamins (B_1_, B_6_, B_9_, and B_12_), vitamin D_3_, vitamin C, and the minerals magnesium, iron, and zinc. These nutrients are recognised as being important for processes such as neurotransmitter function, neuroplasticity, and mood regulation. This nutritional profile strengthens the potential of the beverages as a functional food supplement, especially for patients with limited dietary diversity and an increased risk of nutritional deficiencies. The results emphasise the importance of considering both nutritional value and sensory acceptability when developing protein-rich nutritional products, especially for patients with psychiatric disorders. Further research is required to assess the long-term effects and confirm the potential benefits. It is recommended that future studies extend the intervention period and include a larger and more diverse sample of patients with psychiatric disorders, to evaluate the long-term effects of pea protein isolate drinks on clinical and functional parameters, and to evaluate the effects of long-term use on biochemical parameters, including the lipid profile (total cholesterol and fractions), haematological parameters, total serum protein levels and their fractions, as well as mineral status. Such studies could help establish plant-based protein beverages as a scientifically sound and patient-friendly nutritional intervention in mental healthcare, combining efficacy with acceptability.

## Figures and Tables

**Table 1:** Characterisation of the composition of pea protein isolate beverages with different flavours, g.

List of Products Used in the Beverages	Beverage with Lemon Flavour (V1)	Beverage with Pomegranate–Cranberry Flavour (V2)	Beverage with Blueberry–Vanilla Flavour (V3)	Beverage with Blueberry–Lemon Flavour (V4)	Beverage with Blackcurrant–Apple Flavour (V5)
Pea protein isolate	20	20	20	20	20
Warm water	92	78	82	62	61
Walnut powder	10	-	-	-	-
Vitamins and minerals	1.5	1.5	1.5	1.5	1.5
B12 vitamin	0.000025	0.000025	0.000025	0.000025	0.000025
Magnesium citrate	2	2	2	2	2
Lemon juice	60	-	-	10	-
Apple juice	14	-	-	-	46
Hemp powder	-	10	-	-	-
Pomegranate juice	-	58	-	-	-
Cranberry juice	-	29	-	-	-
Ascorbic acid	-	1	1	-	1
Pine nut powder	-	-	10	10	10
Blueberry juice	-	-	77	78	-
Vanilla extract	-	-	6		-
Blueberry powder	-	-	-	6	-
Inulin	-	-	-	10	-
Blackcurrant juice	-	-	-	-	50
Blackcurrant powder	-	-	-	-	8
Total	200 g	200 g	200 g	200 g	200 g

**Table 2:** Laboratory methods used.

No	Analytical Parameters	Method or Standard
1.	Energy value	Regulation (EU) No 1169/2011 of the European Parliament and of the Council [EN] [35]
2.	Protein	PB-116 ed. 4 of 30.12.2024 [EN] [36]
3.	Amino acids	ISO 13903:2005 (IC-UV); EU 152/2009 (LC-FLD) [37,38]
4.	Fat	PB-286 ed. 2 of 16.01.2025 [EN] [39]
5.	Fatty acid profile	Animal and vegetable fats and oils—Gas chromatography of fatty acid methyl esters. PN-EN ISO 12966-1:2015-01; PN-EN ISO 12966-2:2017-05 except p.5.3 and 5.5; PN-EN ISO 12966-4:2015-07 [40]
6.	Carbohydrates	Regulation (EU) No 1169/2011 of the European Parliament and of the Council [EN] [35]
7.	Total sugars	PB-429 ed. 3 of 29.11.2024 [41]
8.	Dietary fibre	AOAC 991.43:1994 [42]
9.	Moisture	PB-285 ed. I of 26.09.2014 p. 1 [EN] [43]
10.	Ash	PB-285 wyd. I z dn. 26.09.2014 p. 2 [EN] [44]
11.		
12.	Vitamin D_3_ (cholecalciferol)	PN-EN 12821:2009 [45]
13.	Vitamin B_6_	PB-470 ed. I of 11.10.2021 [46]
14.	Vitamin B_7_ (biotin)	PB-326 ed. 3 of 15.04.2025 [47]
15.	Vitamin B_9_ (folic acid)	PB-327 ed. 3 of 15.04.2025 [48]
16.	Vitamin B_12_ (cyanocobalamin)	PB-328 ed. 3 of 15.04.2025 [49]
17.	Vitamin C	PB-135/HPLC ed. II of 15.09.2015 [50]
18.	Zinc (Zn)	PB-36/ICP ed. 8 of 29.12.2022 [51]
19.	Iron (Fe)	PB-223/ICP ed. 4 of 29.12.2022 [52]
20.	Calcium (Ca)	PB-36/ICP ed. 8 of 29.12.2022 [53]
21.	Magnesium (Mg)	PB-36/ICP ed. 8 of 29.12.2022 [54]
22.	Selenium (Se)	PB-223/ICP ed. 4 of 29.12.2022 [55]
23.	Salt as sodium chloride (Nax2.5)	PB-318 ed. 3 of 11.10.2024 [56]

**Table 3:** Nutrient distribution among five pea protein isolate beverages, 100 g^−1^ DW.

Nutrients	V1	V2	V3	V4	V5	RDI
Moisture, g	83.7 ± 4.2	79.3 ± 4.0	78.9 ± 3.9	75.2 ± 3.8	75.5 ± 3.8	
Energy value, kcal	70	81	86	89	97	
Energy value, E% *	7.0	8.1	8.6	8.9	9.7	1840–2510 kcal/d [59]
Fat, g	2.3 ± 0.5	1.4 ± 0.5	1.6 ± 0.5	1.6 ± 0.5	1.7 ± 0.5	
Fat, E% *	6.9	4.2	4.8	4.8	5.1	20–30% [59]
Carbohydrates, g	2.6	7.2	8.0	11.9	9.2	
Carbohydrates, E% *	2.0	5.5	6.2	9.2	7.1	45–60% [59]
Total sugars, g	1.6 ± 0.3	3.6 ± 0.7	6.7 ± 1.3	3.6 ± 0.7	5.2 ± 1.0	
Total sugars, E% *	12.8	28.8	53.6	28.8	41.6	10% [59]
Dietary fibre, g	0.6 ± 0.2	1.1 ± 0.4	0.5 ± 0.2	0.7 ± 0.3	1.4 ± 0.6	25–35 g [60]
Protein, g	9.3 ± 0.9	9.4 ± 0.9	9.9 ± 1.0	9.0 ± 0.9	10.4 ± 0.8	
Protein, E% *	37.2	37.6	39.6	36.0	41.6	10–20% [59]
Salt, g	0.19 ± 0.04	0.19 ± 0.04	0.2 ± 0.05	0.19 ± 0.04	0.2 ± 0.05	5 g [60]

Note: Values are means ± SD (*n* = 5). DW—dry weight. * The daily intake was expressed per 200 g of product.

**Table 4:** Amino acid profile of pea protein isolate beverages with different flavours, g 100 g^−1^ DW.

Amino Acids	V1	V2	V3	V4	V5
Alanine	0.375 ± 0.053	0.372 ± 0.052	0.430 ± 0.060	0.434 ± 0.061	0.468 ± 0.066
Arginine	0.799 ± 0.112	0.733 ± 0.102	0.949 ± 0.133	0.931 ± 0.130	1.01 ± 0.14
Aspartic acid	1.03 ± 0.14	1.02 ± 0.14	1.12 ± 0.16	1.140.16	1.21 ± 0.17
Glutamic acid	1.51 ± 0.21	1.44 ± 0.20	1.67 ± 0.23	1.68 ± 0.24	1.81 ± 0.25
Glycine	0.367 ± 0.051	0.354 ± 0.050	0.405 ± 0.057	0.410 ± 0.057	0.440 ± 0.062
Histidine	0.211 ± 0.029	0.214 ± 0.030	0.234 ± 0.033	0.236 ± 0.033	0.251 ± 0.035
Hydroxyproline *	<0.2	<0.2	<0.2	<0.2	<0.2
Isoleucine	0.395 ± 0.055	0.398 ± 0.056	0.440 ± 0.062	0.445 ± 0.062	0.465 ± 0.065
Leucine	0.720 ± 0.101	0.712 ± 0.100	0.803 ± 0.112	0.814 ± 0.114	0.861 ± 0.121
Lysine	0.592 ± 0.083	0.607 ± 0.085	0.679 ± 0.095	0.682 ± 0.095	0.729 ± 0.102
Ornithine	<0.05	<0.05	<0.05	<0.05	<0.05
Phenylalanine	0.468 ± 0.066	0.466 ± 0.065	0.510 ± 0.071	0.511 ± 0.072	0.551 ± 0.077
Proline	0.370 ± 0.052	0.361 ± 0.051	0.421 ± 0.059	0.432 ± 0.010	0.450 ± 0.063
Serine	0.463 ± 0.065	0.452 ± 0.063	0.524 ± 0.073	0.525 ± 0.074	0.563 ± 0.079
Threonine	0.323 ± 0.045	0.324 ± 0.045	0.357 ± 0.050	0.363 ± 0.051	0.390 ± 0.055
Tyrosine	0.317 ± 0.044	0.304 ± 0.043	0.357 ± 0.050	0.368 ± 0.052	0.383 ± 0.054
Tryptophan	0.0914 ± 0.0091	0.0850 ± 0.0085	0.0986 ± 0.0099	0.0996 ± 0.0100	0.105 ± −0.011
Valine	0.444 ± 0.062	0.449 ± 0.063	0.500 ± 0.07	0.507 ± 0.071	0.541 ± 0.015
Cysteine + Cystine	0.0830 ± 0.0116	0.0860 ± 0.0120	0.102 ± 0.014	0.100 ± 0.014	0.110 ± 0.015
Methionine	0.0940 ± 0.013	0.109 ± 0.015	0.125 ± 0.018	0.125 ± 0.018	0.129 ± 0.018

Note: Values are means ± SD (*n* = 5). DW—dry weight. * Parameter not included in the scope of accreditation.

**Table 5:** Fatty acid profile of pea protein isolate beverages with different flavours, g 100 g^−1^ DW.

Fatty Acids	V1	V2	V3	V4	V5	RDI
Total saturated fatty acids (SFAs)	0.3 ± 0.1	0.2 ± 0.1	0.2 ± 0.1	0.2 ± 0.1	0.2 ± 0.1	
% of the RDI *	2.7%	1.8%	1.8%	1.8%	1.8%	<10% of E% (20–25 g) [63,64]
Total monounsaturated fatty acids (MUFAs)	0.5 ± 0.1	0.3 ± 0.1	0.4 ± 0.1	0.2 ± 0.1	0.4 ± 0.1	
% of the RDI *	3.3%	2.0%	2.7%	1.3%	2.7%	12–20% of E% (25–40 g) [63,65]
Total polyunsaturated fatty acids (PUFAs)	1.6 ± 0.2	0.9 ± 0.1	0.8 ± 0.1	0.9 ± 0.1	1.0 ± 0.1	
% of the RDI *	16.0%	9.0%	8.0%	9.0%	10.0%	6–11% of E% (13–25 g) [63,66]
Trans fatty acids are isomers	0.1 ± 0.1	0.2 ± 0.1	0.1 ± 0.1	0.1 ± 0.1	0.1 ± 0.1	(<2 g) [63,64]
Total omega-3 fatty acids	0.2 ± 0.1	0.2 ± 0.1	0.1 ± 0.1	0.1 ± 0.1	0.2 ± 0.1	≥1.6 g (M)/≥1.1 g (F) [66,67]
Eicosapentaenoic acid (EPA)	0.1 ± 0.1	0.1 ± 0.1	0.1 ± 0.1	0.1 ± 0.1	0.1 ± 0.1	EPA+DHA 1–2 g [67]
Docosahexaenoic acid (DHA)	0.1 ± 0.1	0.1 ± 0.1	0.1 ± 0.1	0.1 ± 0.1	0.1 ± 0.1	
Total omega-6 fatty acids	1.3 ± 0.1	0.7 ± 0.1	0.8 ± 0.1	0.8 ± 0.1	0.8 ± 0.1	
% of the RDI *	20.0%	10.8%	12.3%	12.3%	12.3%	4–8% of E% (9–17 g) [63]
Total omega-9 fatty acids	0.4 ± 0.1	0.3 ± 0.1	0.4 ± 0.1	0.4 ± 0.1	0.4 ± 0.1	
% of the RDI *	4.7%	3.5%	4.7%	4.7%	4.7%	15–20 g/d [65]
α-linolenic acid (ALA)	0.2 ± 0.1	0.2 ± 0.1	0.1 ± 0.1	0.1 ± 0.1	0.2 ± 0.1	
% of the RDI *	30.8%	30.8%	15.4%	15.4%	30.8%	1.1–1.6 g [63,66]

Note: Values are means ± SD (*n* = 5). DW—dry weight. * The daily intake was expressed per 200 g of product.

**Table 6:** Vitamin and mineral content of pea protein isolate beverages, mg 100 g^−1^ DW.

Nutrients	V1	V2	V3	V4	V5	RDI
Vitamin C	42 ± 12.0	461 ± 129	429 ± 120	44 ± 12.0	458 ± 128.0	
% of the RDI *	101.8%	1117.6%	1040.0%	106.7%	1110.3%	75–90 mg [59,68]
Vitamin B_6_	0.71 ± 0.14	0.63 ± 0.13	0.53 ± 0.13	0.65 ± 0.13	0.58 ± 0.12	
% of the RDI *	94.7%	84.0%	70.7%	86.7%	77.3%	1.3–1.7 mg [59,69]
Vitamin B_7_	0.0209 ± 0.0042	0.018 ± 0.0036	0.02 ± 0.0040	0.0167 ± 0.0033	0.0215 ± 0.0043	
% of the RDI *	139.3%	120.0%	133.3%	111.3%	143.3%	0.03 mg [59,70]
Vitamin B_9_	0.195 ± 0.039	0.158 ± 0.032	0.182 ± 0.036	0.157 ± 0.031	0.162 ± 0.032	
% of the RDI *	97.5%	79.0%	91.0%	78.5%	81.0%	0.4 mg [59,71]
Vitamin B_12_	0.008 ± 1.72	0.008 ± 1.61	0.009 ± 1.87	0.007 ± 1.43	0.008 ± 1.66	
% of the RDI *	800.0%	800.0%	900.0%	700.0%	800.0%	0.002 mg [59,72]
Vitamin D_3_	0.001 ± 0.69	0.002 ± 0.77	0.003 ± 0.92	0.003 ± 1.00	0.003 ± 0.93	
% of the RDI *	11.1%	22.2%	33.3%	33.3%	33.3%	0.015 mg–0.020 mg [59,73]
Zinc	6.0 ± 1.38	6.67 ± 1.53	7.32 ± 1.68	7.16 ± 1.65	7.54 ± 1.73	
% of the RDI *	150.0%	166.8%	183.0%	179.0%	188.5%	8 mg [59,74]
Iron	5.88 ± 1.35	5.74 ± 1.32	5.79 ± 1.33	5.54 ± 1.27	6.15 ± 1.41	
% of the RDI *	71.5%	69.6%	70.2%	67.2%	74.5%	15–18 mg [59,75]
Calcium	220 ± 0.05	230 ± 0.06	240 ± 0.06	230 ± 0.06	250 ± 0.06	
% of the RDI *	40.0%	41.8%	43.6%	41.8%	45.5%	1000–1200 mg [59,76]
Magnesium	148 ± 27.0	162 ± 29.0	165 ± 30.0	160 ± 29.00	168 ± 30.0	
% of the RDI *	94.0%	102.9%	104.8%	101.6%	106.7%	310–320 mg [59,77]
Selenium	0.018 ± 0.005	0.018 ± 0.005	0.017 ± 0.005	0.016 ± 0.004	0.018 ± 0.005	
% of the RDI *	65.5%	65.5%	61.8%	58.2%	65.5%	0.055 mg [59,78]
Sodium	76 ± 0.017	77 ± 0.018	80 ± 0.018	77 ± 0.018	79 ± 0.018	
% of the RDI *	7.6%	7.7%	8.0%	7.7%	7.9%	2000 mg [59,79]

Note: Values are means ± SD (*n* = 5). DW—dry weight. * The daily intake was expressed per 200 g of product.

## Data Availability

The data supporting this study’s findings are available upon request from the corresponding author.
